# An MBS-Assisted Femtocell Transmit Power Control Scheme with Mobile User QoS Guarantee in 2-Tier Heterogeneous Femtocell Networks

**DOI:** 10.1155/2013/403978

**Published:** 2013-12-11

**Authors:** Jenhui Chen, Chih-Cheng Yang, Shiann-Tsong Sheu

**Affiliations:** ^1^Department of Computer Science and Information Engineering, School of Electrical and Computer Engineering, College of Engineering, Chang Gung University, Kweishan, Taoyuan 33302, Taiwan; ^2^Department of Communication Engineering, National Central University, Chung-Li 32001, Taiwan

## Abstract

This study investigates how to adjust the transmit power of femto base station (FBS) to mitigate interference problems between the FBSs and mobile users (MUs) in the 2-tier heterogeneous femtocell networks. A common baseline of deploying the FBS to increase the indoor access bandwidth requires that the FBS operation will not affect outdoor MUs operation with their quality-of-service (QoS) requirements. To tackle this technical problem, an *FBS transmit power adjustment* (FTPA) algorithm is proposed to adjust the FBS transmit power (FTP) to avoid unwanted cochannel interference (CCI) with the neighboring MUs in downlink transmission. FTPA reduces the FTP to serve its femto users (FUs) according to the QoS requirements of the nearest neighboring MUs to the FBS so that the MU QoS requirement is guaranteed. Simulation results demonstrate that FTPA can achieve a low MU outage probability as well as serve FUs without violating the MU QoS requirements. Simulation results also reveal that FTPA has better performance on voice and video services which are the major trend of future multimedia communication in the NGN.

## 1. Introduction

Next generation communication networks are expected to provide a function of pervasive network access as well as quality-of-service (QoS) guarantee. The fourth generation mobile communication standard, for example, 3GPP long term evolution-advanced (LTE-A) [1], proposed a *h*-tier heterogeneous network architecture which integrates *h* types of networks for coexisting to achieve this goal. The 2-tier heterogeneous femtocell network is one type of the *h*-tier heterogeneous network architecture, which is composed of one macrocell and several femtocells within the radio coverage of macrocell. The femtocells formed by femto base stations (FBSs) are set by subscribers for the purpose of increasing data access rate in indoor environment [2, 3]. If FBSs are set privately, they are the type of closed subscriber group (CSG) FBSs [4] with which only allow authorized femto users (FUs) to connect. Otherwise, the FBSs belong to the type of full or partial open subscriber group (OSG) FBSs.

Because the macrocell and femtocell networks coexist in the same frequency band, the interference avoidance and mitigation problem dominate the key point of the performance of network coexistence. A femtocell network survey [4] and a femto forum report [5] showed that most used services of mobile users (MUs) are voice calls; more than 50% of voice calls and more than 70% of data traffic occur in indoor environment. Articles [6–8] indicated that if conventional power-control schemes are applied for multimedia traffic without any modification, the system capacity is limited by the traffic with the lowest bit error rate (BER) requirement. Important research studies [6, 7] indicated that voice packets can typically tolerate a BER up to 10^−3^ but are delay sensitive while data packets require a BER below 10^−9^ but are delay insensitive. Wang [8] showed that the medium access control (MAC) protocol design for wireless multimedia network is challenging because multimedia services have heterogeneous BERs and quality-of-service (QoS) requirements. He established the relation between target signal-to-interference-plus-noise ratio (SINR) values and BER requirements by considering the error control schemes and channel fading.  Table 1 shows the BER requirements of different traffic types and their corresponding SINR values.

In femtocell networks, most FBSs are used in indoor environment. In this scenario, several communication properties are specified as follows. First, because most FBSs are usually set indoors and surrounded by concrete walls, the leakage of electromagnetic energy to the outside is degraded significantly. Second, the probability of MU interfered by FBS transmit power (FTP) can be reduced if the FTP is controlled by taking the neighboring MUs (if any) into account. Third, the BER can be improved in indoor environment by adopting an adaptive modulation and coding (AMC) scheme according to radio channel quality, which can be used for heterogeneous femtocell networks deployments [9]. These factors motivate us to mitigate the interference between the macrocell and femtocells by adjusting the FTP and using the AMC scheme with the prerequisite of guaranteeing QoS services [10] of MUs and FUs, which are around FBSs.

The interference problems of the 2-tier heterogeneous femtocell networks are classified into two types: the downlink and uplink interference problems. Many interference management mechanisms were broadly studied such as cochannel femtocell deployment for interference-limited coverage area (ILCA) [11], power control and beamforming disjoint mechanism [12], interference reducing by minimizing the transmit power [13], and self-optimized coverage coordination mechanism [14]. Several spectrum allocation mechanisms were used for spectrum splitting such as preplanned frequency assignment approaches [15], gaming approaches [16, 17], cognitive radio approaches [18, 19], dynamic spectrum allocation and cell association mechanisms [20, 21], and cooperative spectrum allocation mechanism for intercell fairness [22]. In [23, 24], they gave comprehensive studies of analysis and simulation on downlink interference based on a SINR viewpoint because the impact of downlink interference on communications is more serious as compared with the uplink interference. However, none of them considered the QoS properties of service types when they dealt with the problem of mitigating interference.

There are two technical challenges on the interference mitigation. The first challenge is how to adjust the FTP to avoid interfering with the ongoing transmission of neighboring MUs with corresponding QoS parameters. The second challenge is how to choose an appropriate modulation and coding scheme based on the upper bound of adjusted FTP to meet QoS parameters expected by FUs for the services requested.

To conquer these challenges, an FBS transmit power adjustment (FTPA) algorithm which considers MU's QoS requirements is proposed to dynamically adjust FTP for avoiding cochannel interference (CCI) with MUs. FTPA chooses the received SINR of users as the QoS index. The macro-BS (MBS) uses the location information (LI) of neighboring MUs around FBSs to notify corresponding FBSs through the S1 interface (backhaul networks) for CCI mitigation. The overheads of LI forwarding are light because the MBS only notify the nearest MU's LI to the corresponding FBS, and the bandwidth of backhaul is large enough to afford the needed traffic load.

The rest of this paper is organized as follows. The system model of macro- and femto-coexisting networks is introduced in Section 2. A detailed FTPA algorithm is given in Section 3. A simulation scenario and results for evaluation of FTPA algorithm are given in Section 4. Finally, some remarks and future research topics are in Section 5.

## 2. System Model

**Figure 1:**
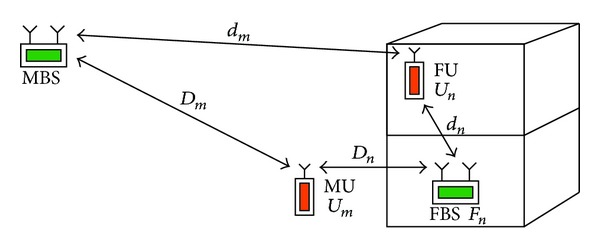
The system model of 2-tier heterogenous femtocell networks.

Figure 1 illustrates a scenario of 2-tier heterogeneous femtocell networks where one MBS and MUs are located in outdoor environment, and the FBSs and FUs are located in indoor environment. Suppose that *N*_*m*_ MUs and *N*_*f*_ FBSs are uniformly distributed in a 3D urban environment [25]. Assume that the probability density function (pdf) of the *x*-nearest FBSs to an MU denoted as *U*_*m*_ (assume that *U*_*m*_ is located at the center of a sphere) follows a homogeneous Poisson point process (HPPP) [26], and is given by

(1) $$\begin{array}{c} P_{X}\left(x,\lambda ,V\right)=\frac{{\left(\lambda V\right)}^{x}}{x!}e^{-\lambda V},\;V=\frac{4\pi r^{3}}{3}, \end{array}$$

where *x* is the number of FBSs in the sphere, *λ* is the density of FBSs, and *r* and *V* are the radius and volume of the sphere. As illustrated in Figure 1, the distance (in meters) between the MBS and the MU is denoted by *D*_*m*_, the distance between the nearest FBS and the the MU is denoted by *D*_*n*_, the distance between the MBS and the FU is denoted by *d*_*m*_, and the distance between the FU and the serving FBS is denoted by *d*_*n*_.

Haenggi [27] showed that the pdf of an Euclidean distance *D*_*n*_ (in meters) between an MU and its *n*th nearest neighboring FBS *F*_*n*_, denoted by *P*_*D*_*n*__(*r*), is distributed according to the generalized gamma distribution as follows:

(2) $$\begin{array}{c} P_{D_{n}}\left(r\right)=\frac{3{\left(4\pi \lambda r^{3}/3\right)}^{n}}{r\Gamma \left(n\right)}e^{-4\pi \lambda r^{3}/3}, \end{array}$$

where Γ(*n*) is the gamma function.

### 2.1. Path Loss

The path loss between a transmitter and receiver in indoor environment is quite different from that in outdoor environment. Based on ITU-R M.1225 slow fading path-loss model [28], the outdoor and pedestrian path-loss, denoted by *L*_*o*_(*D*_*m*_) in dB, from the MBS to an MU *U*_*m*_ is expressed as

(3) Lo(Dm)=δm+10ηm log⁡10(Dm)=30log⁡fc−71+40 log⁡10(Dm),

where *δ*_*m*_ and *η*_*m*_ are the outdoor path-loss constant and exponent of macrocell as shown in Table 2, respectively, and *f*_*c*_ is the central frequency of operating frequency in MHz.

The indoor path loss, denoted by *L*_*i*_(*d*_*n*_), between an FU and its serving FBS *F*_*n*_ with a distance *d*_*n*_ is

(4) Li(dn)=δf+10ηf log⁡10(dn)=37+18.3h((h+2)/(h+1)−0.46)+30 log⁡10(dn),

where *δ*_*f*_ and *η*_*f*_ are the indoor path-loss constant and exponent of femtocell and *h* is the number of floors between *F*_*n*_ and FU in a building. If the FBS and FUs are in the same floor, the value of *h* is equal to zero.

Assume that the FBS is placed inside the house and the radio wave to the MU crosses an external wall of the house. Thus, the indoor to outdoor path loss, denoted by *L*_*x*_(*D*_*n*_), between *F*_*n*_ and *U*_*m*_ is given as

(5) Lx(Dn)=δf+10ηf log⁡10(Dn)=37+18.3h((h+2)/(h+1)−0.46)+δp+30 log⁡10(Dn),

where *δ*_*p*_ is the penetration loss when the radio wave crosses the wall of house. *δ*_*p*_ varies depending on different materials of the wall and we assume that *δ*_*p*_ = 10, 15, and  20 dB in this paper. The interference among femtocells is not considered here because they are separated by concrete walls or obstacles and are set by subscribers in distance [3].

### 2.2. Power Adjustment

Let *ψ*_*m*_^*q*^ be the minimum required SINR threshold (the target SINR) of an MU for achieving one of service types with a QoS index *q*, *q* = 1,2,…, *k*, where *k* is the total number of service types that the MBS provides. Taking Table 1 for example, (*k* = 5), *ψ*_*m*_^1^ = 5.31 stands for the minimal required SINR to achieve voice service and *ψ*_*m*_^5^ = 2.94 stands for the minimal required SINR to achieve data service, and so forth. Because the received SINR of *U*_*m*_, denoted by *ψ*_*m*_(*D*_*m*_), can be simplified by the ratio of the received signal strength from the MBS to its first nearest neighboring FBS *F*_1_ plus noise power [29], we have

(6) ψm(Dm)≈Km−Lo(Dm)−IF1=Km−δm−10ηm log⁡10(Dm)−IF1,

where *K*_*m*_ is the transmit power of the MBS in dB and *I*_*F*_1__ is the interference power from *F*_1_ and is calculated by

(7) IF1=KF1−Lx(D1)=KF1−δf−10ηf log⁡10(D1),

where *K*_*F*_1__ is the FTP of *F*_1_ in dB. In this paper, only the first nearest FBS is considered as the dominating interference source because the difference of signal strength between the first nearest FBS and the tenth nearest FBS is about 15 dB [25]. Finally, considering the background noise to *U*_*m*_, (6) is finalized as

(8) ψm(Dm)≈Km−δm−10ηm log⁡10(Dm)−10 log⁡10·(10(KF1−δf1−10ηf log⁡10(D1))/10+Nm),

where *N*_*m*_ is the noise floor to *U*_*m*_.

**Theorem 1**

To satisfy the QoS requirement of *U*_*m*_, *ψ*_*m*_(*D*_*m*_) ≥ *ψ*_*m*_^*q*^, the maximum allowable FTP of FBS *F*_1_ (i.e., *K*_*F*_1__) follows the inequality

(9) KF1≤Km−δm+δf−10ηm log⁡10(Dm)+10ηf log⁡10(Dn)−ψm+10 log⁡10·(1−Nm10(Km−δm−10ηm log⁡10(Dm)−ψmq)/10).

**Proof**

To satisfy the QoS requirement of the connection which belongs to *U*_*m*_, the MBS selects a modulation and coding rate *M*_*m*_ to transmit packets to *U*_*m*_. Based on the assumption and (8), the required SINR of *U*_*m*_ for *M*_*m*_ must satisfy the condition *ψ*_*m*_(*D*_*m*_) ≥ *ψ*_*m*_^*q*^; then

(10) ψmq≤Km−δm−10ηm log⁡10(Dm)−10 log⁡10·(10(KF1−δf−10ηf log⁡10(D1))/10+Nm).

Rearranging (10) for *K*_*F*_1__, we get

(11) KF1≤Km−δm+δf−10ηm log⁡10(Dm)+10ηf log⁡10(D1)−ψmq+10 log⁡10·(1−Nm10(Km−δm−10 log⁡10(Dm)−ψmq)/10).

Theorem 1 gives the upper bound of the FTP if an MU neighbors the FBS. Taking *K*_*F*_1__ obtained from (9), the received SINR of *U*_*n*_, denoted by *ψ*_*f*_(*d*_*n*_), can be calculated by

(12) ψf(dn)≈KF1−δf−10ηf log⁡10(dn)−10 log⁡10·(10(Km−δm−10ηm log⁡10(dm))/10+Nm).

Suppose that there are *l* different AMC levels supported in the PHY layer. The achievable AMC level depends on the received SINR value *ψ*_*f*_(*d*_*n*_) of user *U*_*n*_. Let *M* = 1,2,…, *l* be the index of AMC levels as shown in Table 3, and *ψ*_*M*_ be the minimal required SINR to achieve the modulation level *M*. Let *M*_*n*_ denote the maximal achievable AMC level for FU *U*_*n*_, and is given by

(13) $$\begin{array}{c} M_{n}≜\left\{M\;|\;\psi_{M}\leq \psi_{f}\left(d_{n}\right)<\psi_{M+1}\right\}, \end{array}$$

where *ψ*_*M*+1_ = *∞* when *M* = *l*. Let *ψ*_*n*_^*q*^ denote the target SINR of an FU *U*_*n*_ for achieving one of service types with a QoS index *q*, *q* = 1,2,…, *k*. According to (12), the FBS can determine an *M*_*n*_ with *K*_*F*_1__ power for QoS level *q* if it satisfies the condition *ψ*_*f*_(*d*_*n*_) ≥ *ψ*_*n*_^*q*^.

## 3. FTP Adjustment Algorithm

Because FTPA is mainly applied in the FBS, some parameters have to be inputted into FTPA prior for calculating the upper bound of FTP.

### 3.1. Outage Probability of MU

Based on (1) and (2), Tseng and Huang [25] showed that the MU outage probability in the cellular networks (i.e., *ψ*(*D*_*m*_) < *ψ*_*m*_^*q*^) is based on a given distance *D*_*m*_, a given FBS density *λ*, and a given target signal-to-interference ratio (SIR). However, in this paper, we consider the SINR as the parameter to obtain the MU outage probability. Hence, the occurring probability of outage events by a given target outage probability Pr_*O*_ (which is the maximal tolerable outage probability of the cellular system) follows the condition

(14) Pr[ψm(Dm)<ψmq] ≈1−exp⁡(−4πλexp⁡⁡(3ζu(Dm))3)<PrO,

where *ζ*_*u*_(*D*_*m*_) is an upper bound value related to *ψ*_*m*_^*q*^ and is given by

(15) ζu(Dm)=  ln⁡(10)10ηf(ψmq−(Km−KF1)+(δm−δf)+10ηm log⁡10(Dm)−10 log⁡10     ·(1−Nm10(Km−δm−10ηm log⁡10(Dm)−ψm)/10)),

where *N*_*m*_ < 10^(*K*_*m*_−*δ*_*m*_−10*η*_*m*_log⁡_10_(*D*_*m*_)−*ψ*_*m*_)/10^. Because the MU outage probability varies with *D*_*m*_, the expected MU outage probability in a macrocell with a radius *R* under a given FBS density *λ*, MBS transmit power *K*_*m*_, and the nearest FTP *K*_*F*_1__, can be obtained by

(16) $$\begin{array}{c} P_{R}=\int_{x=0}^{R}\frac{3x^{2}}{R^{3}}\left[1-\exp\left(\frac{-4\pi \lambda \exp\left(3\zeta_{u}\left(x\right)\right)}{3}\right)\right]dx. \end{array}$$

### 3.2. FTPA with Location Information

Assume that all MUs and FBSs equip with the global positioning system (GPS). Each FBS reports the current location via the backhaul connection (i.e., the S1 interface) to the overlaid MBS when each FBS is set by subscribers. Let *K*_*O*_ denote the operating FTP and let the maximal FTP be the initial value of *K*_*O*_ (e.g., 20 dBm). Each MU reports its current location to the MBS by periodic ranging procedures. The MBS can obtain *D*_*m*_ and *D*_*n*_ derived from the location reported from each *U*_*m*_. The MBS can calculate the received SINR *ψ*_*m*_(*D*_*m*_) of each *U*_*m*_ by applying *D*_*m*_, *D*_*n*_, and *K*_*F*_ into (8). To ensure that QoS services of MUs can be guaranteed, system operators may set a safe SINR difference value *τ* (i.e., *ψ*_*m*_(*D*_*m*_) − *ψ*_*m*_^*q*^ ≥ *τ*) to increase the reliability of these QoS services. When *ψ*_*m*_(*D*_*m*_) − *ψ*_*m*_^*q*^ < *τ*, the MBS notifies the nearest FBS (i.e., *F*_1_) of the MU with the MU's LI after an observation window *T*_*w*_ (a period of time). After receiving the LI, the interfering FBS reduces its FTP to avoid the interference with the MU according to (9). The value of *τ* can be set according to the waiting time period or other effects to guarantee the service continuity of nearby MUs.

Let *I*_*k*_ be a set of MUs which are interfered by FBS *k*, *k* = 1,2,…, *N*_*f*_. If an MU *U*_*m*_ is interfered by FBS *k* (i.e., *ψ*_*m*_(*D*_*m*_) − *ψ*_*m*_^*q*^ < *τ*), the MBS adds the *U*_*m*_ to *I*_*k*_. Notice that an *I*_*k*_ may contains more than one element (i.e., more than one MU is interfered by FBS *k*). To reduce LI notification overheads, the MBS waits a period of *T*_*w*_ to observe the situation of MU interference. If the observed MU is still interfered by the FBS *k* after *T*_*w*_, the MBS notifies the FBS *k* of the current LI of MU. Otherwise, the MU is deleted from *I*_*k*_ and the LI message will not be sent to the FBS *k*.

After *T*_*w*_ elapsing, if *I*_*k*_ ≠ *∅*, the MBS sends LI (the location (*x*, *y*) of each MU and its corresponding QoS level *q*) of all neighboring MUs to the FBS via the backhaul networks. Upon receiving the LI, the FBS *k* executes FTPA to determine the maximal allowable FTP *K*_*F*_ and check whether the use of *K*_*F*_ can satisfy the QoS of its FUs. If no FUs are being served, the FBS *k* discards the LI immediately. The details of FTPA are described as follows.

**Step 1**

Upon receiving the LI, the FBS *k* selects a tuple ((*x*, *y*), *q*) from the LI and determines the maximum allowable FTP *K*_*F*_ according to (9).

**Step 2**

The FBS *k* sets *K*_*O*_ = min⁡(*K*_*O*_, *K*_*F*_). Repeat Step 1 until all the tuples of the received LI are treated.

**Step 3**

The FBS *k* selects one FU from served FUs to obtain *d*_*n*_ and *d*_*m*_ and applies the obtained *K*_*O*_ (treated as *K*_*F*_1__), *d*_*n*_, and *d*_*m*_ into (12) to obtain *ψ*_*f*_(*d*_*n*_).

**Step 4**

If *ψ*_*f*_(*d*_*n*_) ≥ *ψ*_*n*_^*q*^, the FBS *k* selects the highest achievable AMC level *M*_*n*_ according to (13). Otherwise, the FBS *k* uses *K*_*O*_ with the lowest AMC level to serve *U*_*n*_.

**Step 5**

Repeat Steps 3 and 4 until all served FUs are visited.

When an MU moves close to an FBS (the MBS sends the LI message to the FBS), the FBS executes FTPA to reduce its FTP to avoid interfere with the MU. The FTP reduction of the FBS is temporal. The FBS will return its *K*_*O*_ to the maximal power when the neighboring MUs move away from the FBS's radio coverage. To ensure the FBS can return its maximal FTP, the MBS notifies the FBS of the MU leaving a message once the neighboring MU of the FBS leaves the radio coverage of the FBS (i.e., *ψ*_*m*_(*D*_*m*_) − *ψ*_*m*_^*q*^ ≥ *τ*).

## 4. Simulation Results

This section presents a series of simulations for performance evaluation of FTPA. The scenario of simulation consists of one MBS and multiple FBSs around the MBS located in a sphere area with a radius of 424 meters. Three types of QoS are adopted in the simulation. They are the voice type (*ψ* ^*q*^ = 5.31 dB), the CBR video type (*ψ* ^*q*^ = 9.32 dB), and the data type (*ψ* ^*q*^ = 2.94 dB), for example, email or control messages. To focus on the effect of adjusting FTP on MU's QoS achievement, the antenna gain is not considered in this simulation. The coverage radius varies by different QoS types because different QoS types require different SINR values. Thus, in the simulation, the order of coverage radius of voice, CBR video, and data is data > voice > CBR video.

The number of FBSs is adjusted and controlled by varying the FBS density (*λ*) which ranges from 10^−6^ to 10^−8^ m^−3^ to observe the impact of femtocell networks on the macrocell network. The communication radius of the femtocell is 20 meters. All the FBSs are OSG FBSs, and the location of FBSs is randomly deployed in the macrocell. The path loss models follow the definitions and descriptions in Section 2.1. All FBSs are in the indoor environment. To evaluate the effect of FTP adjustment on MU interference, three types of scenarios (i.e., different thicknesses of walls are considered *δ*_*p*_ = 20,15, 10 dB) are adopted in this simulation. The parameter *δ*_*p*_ = 20 dB indicates that FBSs are deployed in the concrete buildings which degrade the signal strength significantly. Contrarily, *δ*_*p*_ = 10 dB represents the wooden houses in which signals can easily pass through the wall. The SINR safe difference value *τ* is set as 0. The height of each MU is 1.5 m. Other simulation parameters are shown in Table 4.

The numerical result (Num) of the MU outage probability obtained from (16), the approximation (Approx) approach based on the dominating interference source only [25], and FTPA are simulated for performance comparison. This simulation does not consider the interfemtocell interference. Each simulation result is obtained by calculating the average for 10,000 random scenarios for all experiments. Four performance criteria are measured to evaluate the performance.

Consider the following.

- MU outage probability (*P*_*R*_): the probability that the received SINR of *U*_*m*_ cannot satisfy the QoS requirement *ψ*_*m*_^*q*^ of *U*_*m*_.
- Mean MU SINR (*ψ*_*m*_(*D*_*m*_)): the mean received SINR of MUs.
- System capacity: the capacity achieved by the macrocell and femtocells in the overlaid area.
- MU data rate (Mbps): the mean data rate (mega bits per second) per each MU. This value is obtained by Shannon's formula [31].

**Figure 2:**
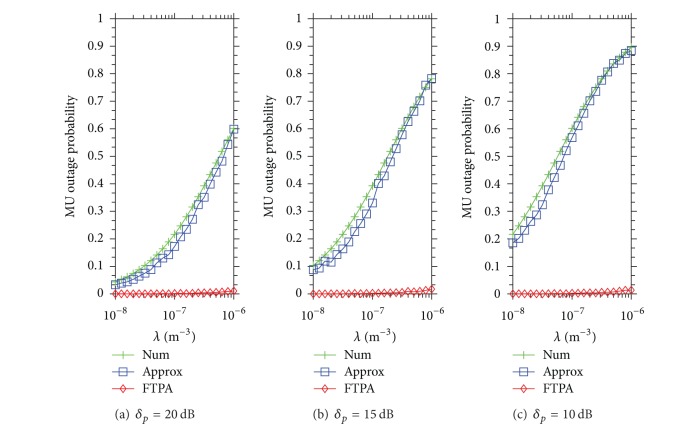
MU outage probability caused by Approx and FTPA versus the density of FBSs development when the QoS type of each MU is voice (*ψ*_*m*_^1^ = 5.31 dB).

**Figure 3:**
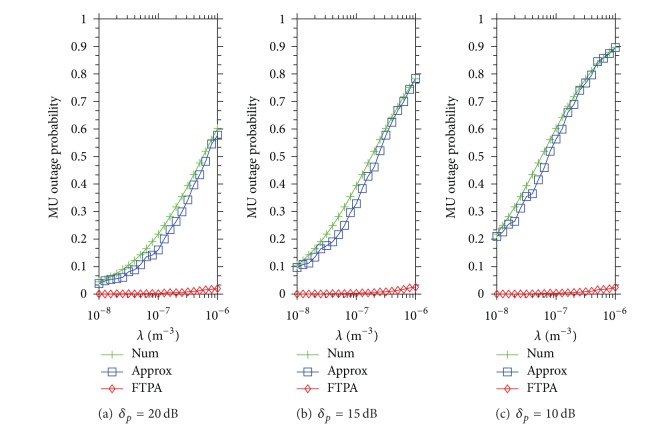
MU outage probability caused by Approx and FTPA versus the density of FBSs development when the QoS type of each MU is CBR video (*ψ*_*m*_^3^ = 9.32 dB).

**Figure 4:**
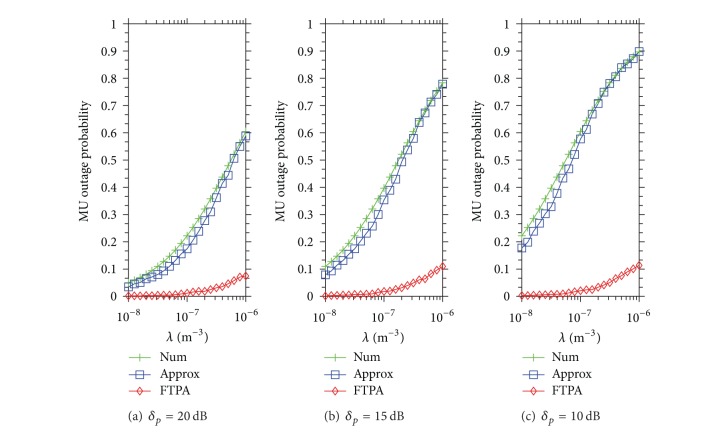
MU outage probability caused by Approx and FTPA versus the density of FBSs development when the QoS type of each MU is data (*ψ*_*m*_^5^ = 2.94 dB).

Figures 2, 3, and 4 demonstrate the MU outage probability caused by using Approx and FTPA versus the density of FBSs development under three different wall material scenarios. The Num is the upper bound of MU outage probability caused by coexisting FBSs where FBSs do not apply the FTP adjustment scheme.  Figure 2 shows the MU outage probability of Num, Approx, and FTPA under three types of wall penetration loss (*δ*_*p*_ = 20,15, 10 dB) when the QoS type of all FBSs is voice (*ψ*_*m*_^*q*^ = 5.31 dB). The MU outage probability of Num and Approx raises quickly as the FBS density increases under different types of penetration loss. The phenomenon becomes more obvious when *δ*_*p*_ is small. Taking *δ*_*p*_ = 20 dB in Figure 2(a); for example, the MU outage probability of Num and Approx is about 0.03 when *λ* = 10^−8^, and raises up to 0.6 when *λ* = 10^−6^. The MU outage probability of Num and Approx raises more significantly when *δ*_*p*_ is smaller as shown in Figures 2(b) and 2(c).  Figure 2(b) demonstrates that the MU outage probability raises from 0.11 to 0.8 as the FBS density raises from 10^−8^ to 10^−6^. The MU outage probability is up to 0.9 when *δ*_*p*_ = 10 dB and *λ* = 10^−6^, as shown in Figure 2(c). This is because the leaked FTP is higher when *δ*_*p*_ is lower, and thus leads to MU suffering higher unwanted interference power.

Contrarily, the MU outage probability achieved by FTPA is very low (*P*_*R*_ < 0.02) even when the FBS density is high (*λ* = 10^−6^) and the penetration loss is low (*δ*_*p*_ = 10 dB). This result shows that FTPA decreases the interfering FTP to satisfy the neighboring MU QoS requirements and then reduces the MU outage probability significantly. For instance, *P*_*R*_ = 0.0138 when *λ* = 10^−6^ in Figure 2(c). From the observation of these results, it shows that the outage probability can be controlled easily by only considering the dominating interference because the second interference is not strong enough to interfere with the nearby MUs.

Figure 3 demonstrates the MU outage probability when the QoS service type is CBR video. The simulation result is similar to that shown in Figure 2. The MU outage probability is up to *P*_*R*_ = 0.6 when *δ*_*p*_ = 20 dB, *P*_*R*_ = 0.8 when *δ*_*p*_ = 15 dB, and *P*_*R*_ = 0.9 when *δ*_*p*_ = 10 dB. Meanwhile, FTPA remains in low *P*_*R*_ even in high FBS density *λ* = 10^−6^ (*P*_*R*_ = 0.025). Similarly, Figure 4 demonstrates the MU outage probability when the QoS type is data. It shows that the MU outage probability achieved by FTPA reaches 0.1 when *λ* = 10^−6^ and *δ*_*p*_ = 10 dB. The reason that the MU outage probability increases more obviously than the case of *ψ*_*m*_^1^ and *ψ*_*m*_^3^ is as follows. First, because the required SINR of data service *ψ*_*m*_^5^ = 2.94 is lower than *ψ*_*m*_^1^ = 5.31 and *ψ*_*m*_^3^ = 9.32, the FBS uses higher FTP to serve its FUs (according to (9)). As a result, MUs are interfered by FBSs highly. Second, the coverage radius of data service is longer due to the lower *ψ*_*m*_ requirement. Thus, MUs located in the boundary of the transmission range get lower SINR (due to path loss) and then easily interfered by neighboring FBSs. These results show that FTPA can satisfy all types of QoS of MU in all cases because the maximal MU outage probability is lower than 0.1 which is a *P*_*R*_ theorem bound for system operation.

**Figure 5:**
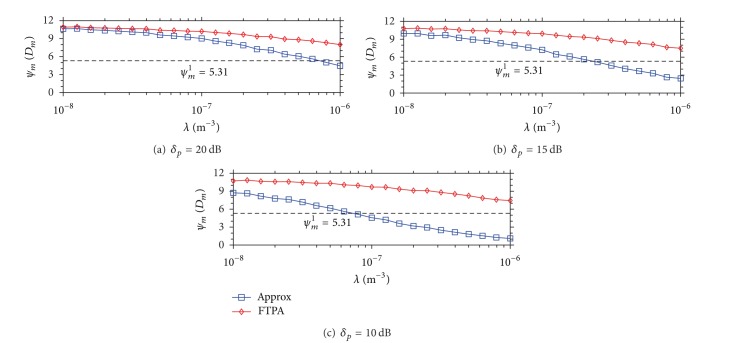
Mean SINR of each MU when the QoS type is voice (*ψ*_*m*_^1^ = 5.31 dB).

Figure 5 shows the mean MU SINR when the QoS type of all MUs is voice under different types of penetration loss. It shows that the density of FBS deployment has to decrease when *δ*_*p*_ decreases. When *δ*_*p*_ = 20 dB, as shown in Figure 5(a), the feasible FBS density with the QoS requirement of voice (*ψ*_*m*_^*q*^ = 5.31 dB) must be lower than 10^−6.2^. This situation is much more obvious when *δ*_*p*_ is lower; that is, *δ*_*p*_ = 15 or 10 dB. The feasible FBS density is lower than 10^−6.7^ when *δ*_*p*_ = 15 dB (see Figure 5(b)) and is lower than 10^−7.2^ when *δ*_*p*_ = 10 dB (see Figure 5(c)). This is because lower *δ*_*p*_ leads to much leaked FTP from nearby FBSs and causes higher interference with MUs. Consequently, the density of FBS development must decrease.

However, as shown in Figure 5, FTPA takes advantage of FTP adjustment to guarantee the QoS requirement of nearby MUs from being interfered by FBS. As we can see from the results, FTPA maintains higher *ψ*_*m*_(*D*_*m*_) than the threshold *ψ*_*m*_^1^ in all FBS densities. The mean SINR of FTPA decreases slightly as the FBS density increases, and it is always higher than the threshold *ψ*_*m*_^1^ even when the FBS density is in *λ* = 10^−6^. No matter what the value of *δ*_*p*_ is, the FTPA can still guarantee the mean SINR for MUs about 8 dB when *λ* = 10^−6^.

**Figure 6:**
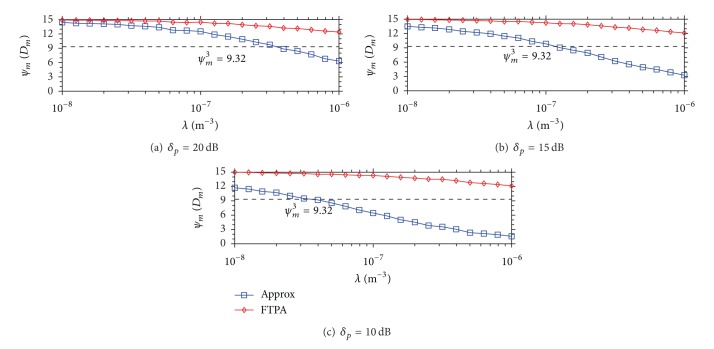
Mean SINR of each MU when the QoS type is CBR video (*ψ*_*m*_^3^ = 9.32 dB).

**Figure 7:**
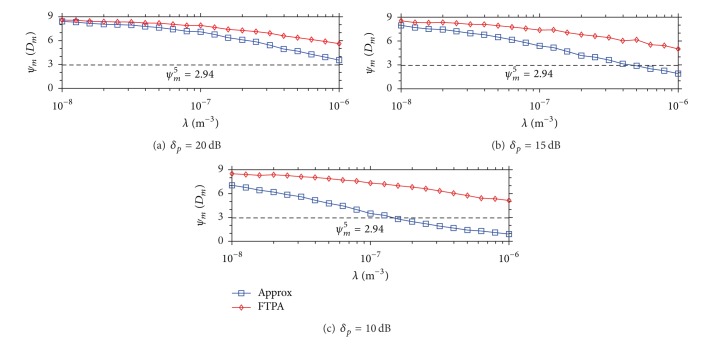
Mean SINR of each MU when the QoS type is data (*ψ*_*m*_^5^ = 2.94 dB).

When the QoS type is CBR video, the feasible FBS density with the mean SINR is shown in Figure 6. To meet the higher QoS requirement, the feasible FBS density of Approx is lower than 10^−6.5^. As compared with the QoS type of voice, the feasible FBS density (i.e., applying the video service) degrades from 10^−6.2^ to 10^−6.5^ (see Figure 6(a)).  Figure 7 reveals the similar results with that of Figures 5 and 6. Based on these results, a fact can be concluded that FTPA can maintain the mean MU SINR to guarantee the QoS requirement of MU as well as increase the density of FBS development and thus increase the overall system capacity (we discuss it in the following).

To investigate the system capacity achieved by FTPA and Approx approaches, the system capacity of each femtocell is normalized as 1 if the FBS can provide services with a modulation and coding rate of QPSK-1/2 for FUs within 20 m (*D*_*n*_ = 20). The AMC scheme is adopted in the simulation (see Table 3). That is, if the received SINR of an FU located at the point of 20 m from the FBS can support the modulation and coding of 16QAM-1/2, the system capacity of the femtocell becomes 2, and so forth (i.e., 64QAM-1/2 is equal to 3).

**Figure 8:**
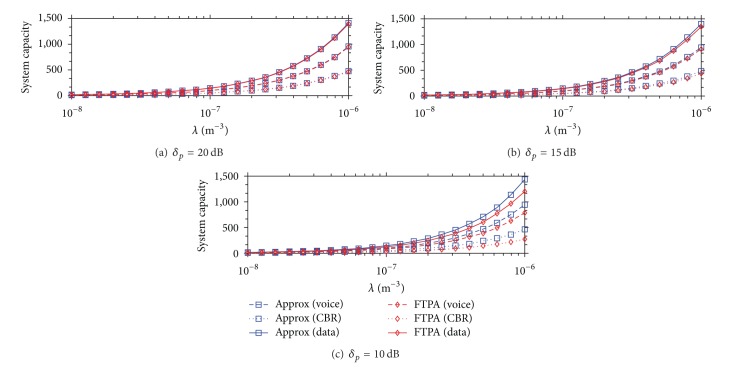
Aggregate system capacity achieved by FTPA and Approx versus the FBS deployment density under different *δ*_*p*_.

Figure 8 shows the aggregate system capacity under different densities of FBS deployment. The QoS type of voice, CBR video, and data traffic dominate 33% of the total traffic load in the simulation separately. Although increasing the number of FBSs can increase the aggregate system capacity, the MU outage probability will also increase as discussed above. FTPA can decrease the MU outage probability by adjusting each FTP. However, decreasing each FTP results in decreasing the femtocell capacity.  Figure 8(c) shows the consequence of decreasing FTP caused by FTPA that the aggregate system capacity is lower than that of Approx. However, the consequence becomes unapparent when *δ*_*p*_ increases (e.g., concrete walls). These results indicate how FTPA takes advantage of decreasing the femtocell capacity to satisfy the MU QoS requirement.

**Figure 9:**
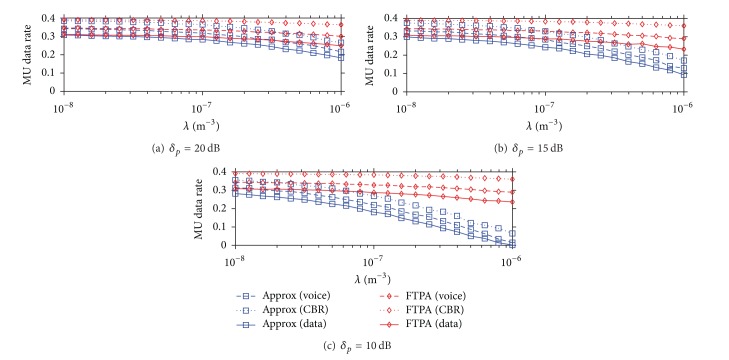
MU data rate achieved by FTPA and Approx versus the FBS deployment density under different *δ*_*p*_.

Figure 9 shows the MU data rate achieved by FTPA and Approx approaches. Although the difference of aggregate system capacity between FTPA and Approx becomes larger when *δ*_*p*_ decreases and *λ* increases (see Figure 8), the MU data rate decreases very quickly (see Figure 9). This means that the MU QoS is sacrificed to increase the femtocell capacity. However, because FBSs are deployed in the indoor environment, the number of FUs served by one FBS is small (i.e, only 1 or 2 FUs). As a result, FUs may not fully utilize the whole femtocell capacity, and the higher MU outage probability is caused (e.g., more than half MUs cannot obtain access service when *λ* = 10^−6^ as shown in Figures 2, 3, and 4). This result provides a strong proof that adjusting FTP to guarantee MU QoS is a feasible solution in the 2-tier heterogeneous femtocell networks.

## 5. Conclusion

In this study, FTPA is proposed to overcome the interference problem among the MUs and FBSs in the 2-tier heterogeneous femtocell networks. The MU QoS requirement cannot be guaranteed if the interfering FBSs do not reduce the FTP. Considering that the MU locations and penetration loss of different wall materials to assist the FTP adjustment will help the FBS reduce the interfering probability with the nearby MUs as well as provide the indoor network access for FUs. Simulation results give the evidence that FTPA achieves lower MU outage probability, maintains mean MU SINR (i.e., MU data rate), and does not affect the mean FU bandwidth. FTPA is easy to be implemented in the fourth generation networks and meets the trend of NGN in which multimedia applications (voice and video) are the major traffic. In the future work, the femtocell capacity can be improved further if the resource scheduling for MUs is provided to dynamically adjust the FTP. Taking MU handover mechanism to avoid MU interference as well as increase the access bandwidth or offload the loading of macrocell into account is another emerging problem to solve in NGN.

## Figures and Tables

**Table 1:** Parameters of different traffic types [8].

Index	Service type	BER	SINR	Timeout	Characteristic
1	Voice	10^−3^	5.31	2 frames	Delay sensitive Talkspurt and silence
2	Audio	10^−4^	7.31	6 frames	Delay sensitive Stream
3	CBR video	10^−5^	9.32	5 frames	Delay insensitive Constant bit-rate
4	VBR video	10^−6^	11.34	4 frames	Delay insensitive Variable bit-rate
5	Data	0	2.94	∞	Delay insensitive Variable size

**Table 2:** Path loss parameters [28].

Path loss	*δ*_*m*_ or *δ*_*f*_	*η*_*m*_ or *η*_*f*_
*L*_*o*_(*D*_*m*_)	30 log⁡ *f*_*c*_ − 71	4
*L*_*i*_(*d*_*n*_)	37 + 18.3*h* ^((*h*+2)/(*h*+1)−0.46)^	3
*L*_*x*_(*D*_*n*_)	37 + 18.3*h* ^((*h*+2)/(*h*+1)−0.46)^ + *δ*_*p*_	3

**Table 3:** Modulation and coding parameters [30].

Level (*M*)	Modulation	Required SINR (*ψ*_*M*_)
1	QPSK (1/2)	5 dB
2	QPSK (3/4)	8 dB
3	16-QAM (1/2)	10.5 dB
4	16-QAM (3/4)	14 dB
5	64-QAM (1/2)	16 dB
6	64-QAM (2/3)	18 dB
7	64-QAM (3/4)	20 dB

**Table 4:** System parameter for simulation.

Parameter	Value
Center frequency	2.5 GHz
Bandwidth	10 MHz
FFT size	1024
Macrocell radius	400 m
FBS radius	20 m
Total BS TX power	46 dBm
Total FBS TX power	20 dBm
Antenna gain	0 dBi
Penetration loss	10, 15, 20 dB
MU distribution	Uniform
Number of MUs	100
Thermal noise	−92.974 dBm
